# Arnold-Chiari Malformation Type I Evident After a Streptococcal Throat Infection in a Young Female: A Rare Case

**DOI:** 10.7759/cureus.42024

**Published:** 2023-07-17

**Authors:** Maryam Shahab, Tamanna Nazir, Sumaria Nazir

**Affiliations:** 1 Internal Medicine, Central Park Physicians, New York, USA; 2 Internal Medicine, Hayatabad Medical Complex, Peshawar, PAK

**Keywords:** pain, syncope, streptococcal throat infection, epilepsy, arnold-chiari malformation

## Abstract

Arnold-Chiari malformation (ACM) is a rare neurological developmental disorder that presents at birth. No such cases have been reported in support of microbial infections causing Chiari malformation, yet there is evidence of how microorganisms can lead to brain abscess, brain empyema, and meningoencephalitis. We present a 23-year-old young woman with progressive back and leg pain after a streptococcal throat infection, followed by a single episode of syncope. Radiographs of the spine revealed a mild reversal of cervical spine curvature and minimal levocurvature of the lumbar spine. Magnetic resonance imaging of the brain showed herniation of the cerebral tonsils into the foramen magnum, which suggested the diagnosis of ACM type I. Neurosurgery was recommended for posterior fossa decompression, but the patient was reluctant to undergo the procedure. This is a rare case of ACM symptomology that became evident only after a streptococcal throat infection in a young adult female.

## Introduction

Arnold-Chiari malformation (ACM) is the pathological herniation of the cerebellar tonsils through the foramen magnum into the cervical canal [1]. ACM type I, being the most common type, consists of a downward tonsillar descent of at least 5 mm or more [2]. ACM type II is characterized by caudal herniation of the brainstem, comprising the inferior vermis, pons, and medulla, in addition to elongation of the fourth ventricle and cerebellar tonsillar displacement [3]. ACM I is usually asymptomatic until adulthood [4] and is an incidental finding [5]. However, this complex and challenging condition causes autonomic dysfunction due to cerebrospinal fluid obstruction and consequent syringomyelia [1,2]. The semiology of events includes chronic head and neck pain, dizziness, visual and sensory symptoms, postural hypotension and tachycardia, and syncope [6]. Previous studies reported that structural underdevelopment may be implicated in the etiology of ACM [5]. In addition, familial history and genetic defects can also be possible etiologies for ACM [2].

Streptococcal throat infection (strep throat) is caused by group A beta-hemolytic *Streptococcus pyogenes* [7]. This bacterial cause of pharyngitis is frequently associated with post-streptococcal glomerulonephritis, acute rheumatic fever, and rheumatic heart disease [7,8]. Although a few case reports have been published on the possible association between strep pharyngitis and intracranial infections [9-11], the role of microbial infection in ACM is not clear. To date, several cases have reported patients with brain empyema, brain abscess, and meningoencephalitis who have been infected with strep throat, but no data have been published on the onset of clinical manifestations of ACM after strep pharyngitis.

We report the first case of ACM type I that became evident after a streptococcal throat infection.

## Case presentation

A previously healthy 23-year-old right-handed female presented via the outpatient department on December 8, 2021, for the account of chronic bilateral foot arch pain, paroxysmal diffuse spine, and neck pain for nine months. The paroxysmal event started in March 2021 in the setting of a streptococcal throat infection, which was confirmed with a positive strep test. A few days after recovering from strep throat, she woke up having sharp pain in her right leg spreading to her right shin, which moved to involve her left leg as well. The everyday pain gradually spread to involve the entire spine and hips. The pain increased in intensity with everyday movements and ambulation. The pain was dull in nature with a score of 3 on a scale of 1-10 and shooting pain with a score of 8 on a scale of 1-10. There was associated intermittent numbness in both feet and tailbone. She reported using meloxicam 7.5 mg, which helped in the past but showed minimal benefit now. Worsening fatigue and intermittent positional dizziness triggered by vertical position and walking were reported, in addition to blurry vision of bilateral eyes, numbness and tingling sensation of her legs, and memory difficulties with remembering tasks associated with pain. No associated muscle weakness, vertigo, headache, seizure, and other constitutional symptoms were reported.

She was diagnosed with anemia, anxiety, and depression in the past while her surgical history was unremarkable. She had known allergies to cat hair, dust mites, and penicillin. She was an active cigarette smoker with a 1.25-pack year for the past five years. No known family history of epilepsy was present. The patient reported head trauma at the age of 17 years, when she fell off a horse, hit her head, and lost consciousness. No known infections of the brain were present. No history of generalized tonic-clonic seizures was noted.

At presentation, she was alert, awake, and oriented to place, time, and person. She was in mild distress, emotionally labile, and looked anxious. Deep tendon reflexes were symmetrical, but brisk in both arms and legs. Natural gait and tandem gait were unsteady. Heel-walking and toe-walking were difficult to perform. There was some tenderness to palpation over the paravertebral points of the cervical, thoracic, and lumbar spine. The range of motions in the spine was limited. The rest of the clinical examination was insignificant.

**Figure 1:**
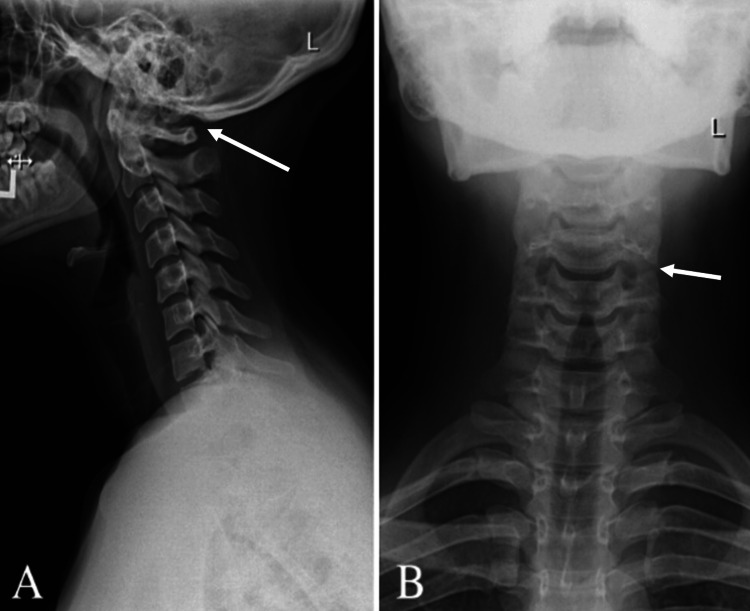
Radiograph of the cervical spine (A) lateral view and (B) anterior-posterior view demonstrating maintained vertebral body heights, disc spaces, and facet joints with mild reversal of the usual cervical lordosis without spondylolisthesis and maintained atlantodental articulation.

**Figure 2:**
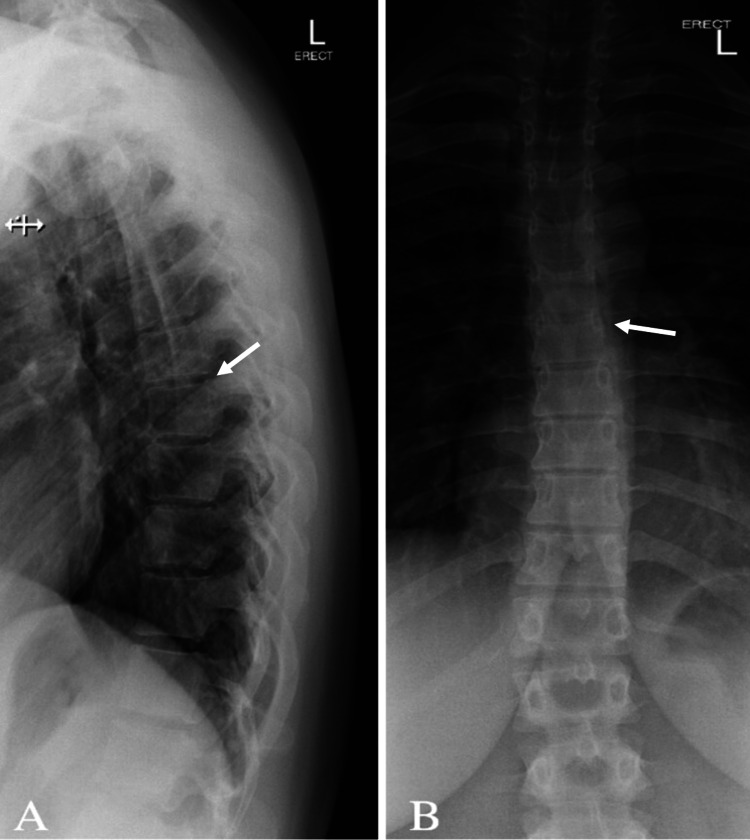
Radiograph of the thoracic spine (A) lateral view and (B) anterior-posterior view demonstrating 12 thoracic vertebrae. Maintained vertebral body heights and disc spaces. No spondylolisthesis. Patent neural foramina.

**Figure 3:**
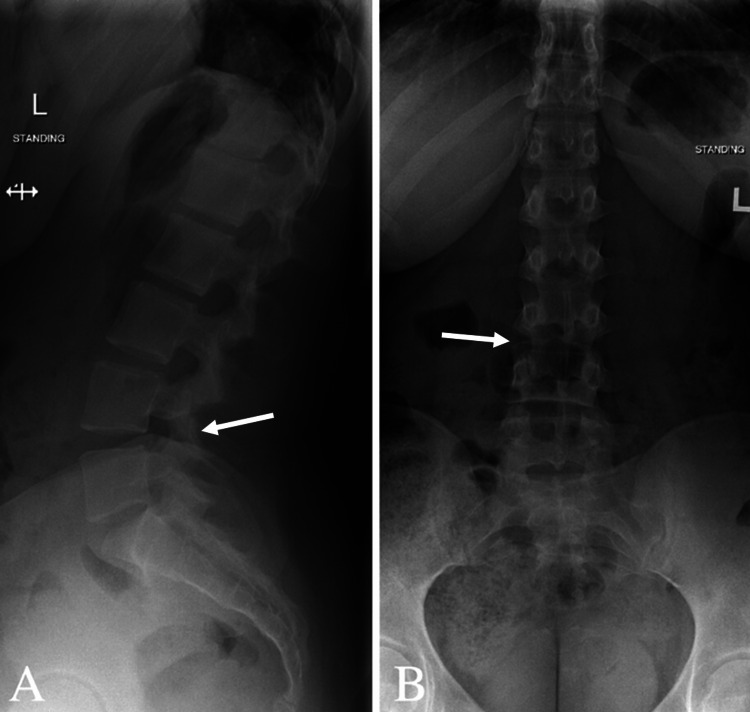
Radiograph of the lumbar spine (A) lateral view and (B) anterior-posterior view demonstrating five lumbar vertebrae. Maintained vertebral body heights and disc spaces. No spondylolisthesis. Patent neural foramina and minimal levocurvature of the lumbar spine.

Routine blood tests were within normal limits. Initial assessments included X-rays (XR) of the cervical spine, thoracic spine, and lumbar spine. XR of the cervical spine showed vertebral body heights, disc spaces, and facet joints (Figure 1). There was a mild reversal of the usual cervical lordosis without spondylolisthesis. XR of the thoracic and lumbar spine showed minimal levocurvature of the lumbar spine (Figures 2, 3).

The patient returned to the outpatient clinic for an episode of syncope that occurred on January 23, 2022, after standing for approximately 20 minutes while smoking a cigarette. She suddenly developed nausea while talking to a friend. She felt ringing in her ears while walking to the front door and her vision started to dim from the periphery followed by a loss of consciousness for less than one minute. She was told her face turned gray before she collapsed. She denied hitting her head because her friend caught her. After returning to her baseline, she felt reduced sensations in her left arm for a few days and developed paresthesia in all her extremities that night. She denied convulsions, tongue biting, and incontinence during the event; however, she reported headaches, nausea, drowsiness, and persistent weakness and numbness after the event. She had trouble understanding what her roommates were saying after the syncopal episode and did not feel recovered until one to two hours later. This was a single isolated event that had not recurred; she denied any cardiac symptoms with or without exertion. She was referred to a cardiologist and neurologist. An electrocardiogram (EKG), ultrasound (US) duplex carotid artery bilateral, and a routine electroencephalogram (EEG) awake and drowsy were carried out.

**Figure 4:**
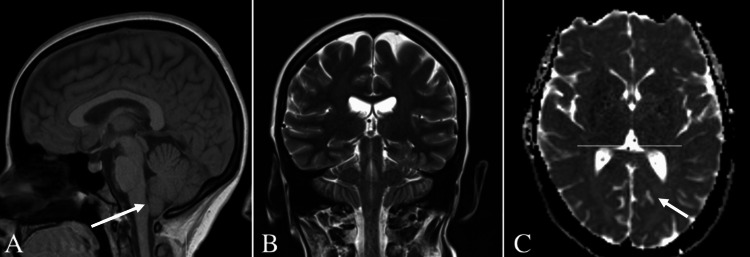
MRI of the brain without contrast of Arnold-Chiari malformation I. (A) Sagittal view demonstrates the herniated cerebellar tonsils descending below the level of the foramen magnum. (B) Coronal view and (C) axial view illustrate caudal downward tonsillar descent of more than 5 mm.

The cardiovascular examination was unremarkable. Her EKG was notable for possible left atrial enlargement that was likely false positive. US duplex carotid arteries were only significant for the mean carotid intima-media thickness (CIMT) test in this patient being low, with less than the 25th percentile of the population value adjusted for age and gender. An echocardiogram (echo) was performed to rule out structural heart diseases as a cause of syncope, which later proved to be normal. A multi-planar, multi-sequential magnetic resonance imaging (MRI) of the brain without intravenous (IV) contrast revealed mild inferior cerebellar tonsillar ectopia with a slightly pointed configuration of the cerebellar tonsils, suggesting mild Chiari I deformity (Figure 4). The patient was instructed to follow up for further workup.

A 21-channel EEG recording using the international 10-20 system was performed, utilizing a NicOne system (Natus, Middleton, Wisconsin), on April 15, 2022. The EEG study was normal in both the awake and drowsy states, neither refuting nor supporting the diagnosis of epilepsy. The patient underwent video EEG (VEEG), which was normal. A single clinical event occurred on April 29, 2022, at 18:12, during which time she reported "pain in her legs." She relayed that this was a typical event as described in the patient history, also associated with other symptoms such as confusion, blurry vision, and drowsiness. She reported that these other symptoms are always preceded by pain in her legs, which typically begins on bilateral anterior shins. No ictal epileptic EEG correlation was appreciated. Orthostatic blood pressure (BP) was checked and with one set there was an increase of 30 in her heart rate upon standing without a decrease in BP, suggesting postural orthostatic tachycardia syndrome (POTS). She has not had a bout of loss of consciousness; however, on two occasions, she became lightheaded upon standing. A tilt table test was advised.

A neurosurgery consultation was done, and posterior fossa surgical decompression was advised. However, the patient is reluctant to go through this procedure due to the uncertainty of benefits and repeat surgeries needed.

## Discussion

We presented a case of ACM type I associated with strep throat in a 23-year-old female. The patient developed a sore throat and after about a week presented subsequently with manifestations of ACM, including backache, syncope, and myelopathy. Recent studies have shown some clinical situations caused by untreated bacterial infections as the underlying etiology in patients with rare neurological diseases.

*Streptococcus pyogenes* is a group A, beta-hemolytic, gram-positive cocci in chains that cause pharyngitis. This acute bacterial infection is associated with impetigo, scarlet fever, and post-streptococcal glomerulonephritis. We present a patient who showed clinical signs of ACM a few days after contracting strep throat. Randhawa et al. reported a case of *Streptococcus* evolving into meningoencephalitis [12]. They found that *Streptococcus* triggered an inflammatory response and resulted in the development of a neurological condition. There are no published data on the association of *Streptococcus* with ACM, but several studies have linked this species with other neurological complications such as Sydenham’s chorea [13], meningitis [9], abscesses [11], empyema [10], and sepsis-associated encephalopathy [9,12].

Chiari malformation, which is associated with bony anomalies at the craniovertebral junction, constitutes one of the primary causes of syringomyelia [1] and occipital hypoplasia [2]. This phenomenon of events causes the posterior fossa to be stuffed to capacity, and the cerebellum and hindbrain herniate through the foramen magnum. The common presentation includes tonsillar ectopia, ocular disturbances, gait ataxia, sensory loss, motor weakness, and cerebellar signs [1,5]. A rare and catastrophic event is sudden death [6], most likely due to medullary compression resulting in hypoxia. A few medical conditions that increase the risk of ACM are Williams syndrome, achondroplasia, Albright hereditary osteodystrophy, velocardiofacial syndrome, aqueductal stenosis, and Klippel-Feil syndrome [2]. Differential diagnoses include but are not limited to incidental tonsillar ectopia, intracranial hypotension [5], basilar migraine [6], astrocytoma, and meningocele. However, the pathophysiology is yet not clear of ACM post-streptococcal pharyngitis.

## Conclusions

Although single-case observations have limitations, the presented case report demonstrates the possible involvement of group A streptococcal throat infection in the presentation of ACM. This case study being an addition to the literature, future case reports and systematic studies, including larger cohorts and rigorous methodologies, may establish a stronger evidence base for this association. More studies and cases of infectious disorders are needed to declare themselves after bacterial infections such as strep throat.
